# 5-Fluorouracil: Mechanisms of Resistance and Reversal Strategies

**DOI:** 10.3390/molecules13081551

**Published:** 2008-08-05

**Authors:** Ning Zhang, Ying Yin, Sheng-Jie Xu, Wei-Shan Chen

**Affiliations:** 1 Department of Orthopaedics, 2nd Affiliated Hospital, School of Medicine, Zhejiang University, #88 Jiefang Road, Hangzhou, 310009, P.R. China; E-mail: zhangning98@gmail.com; 2 Institute of Clinical Research, Sir Run Run Shaw Hospital, School of Medicine, Zhejiang University, #3 East Qingchun Road, Hangzhou, 310016, P.R. China; E-mails: dy1225@gmail.com; xusj@zju.edu.cn

**Keywords:** 5-Fluorouracil, mechanism, resistance, microarray, therapeutic strategies

## Abstract

The purpose of this work is to review the published studies on the mechanisms of action and resistance of 5-fluorouracil. The review is divided into three main sections: mechanisms of anti-tumor action, studies of the resistance to the drug, and procedures for the identification of new genes involved in resistance with microarray techniques. The details of the induction and reversal of the drug resistance are also described.

## Introduction

5-Fluorouracil (5-FU) is still a widely used anticancer drug. Since 1957, it has played an important role in the treatment of colon cancer and is used for patients with breast and other cancers, like those of the head and neck [1].

5-FU is a heterocyclic aromatic organic compound with a structure similar to that of the pyrimidine molecules of DNA and RNA; it is an analogue of uracil with a fluorine atom at the C-5 position in place of hydrogen [2]. Only one crystal structure is reported in the literature for pure 5-FU, in which the compound crystallizes with four molecules in the asymmetric unit and the molecule adopts a hydrogen-bonded sheet structure [3,4]. Due to its structure, 5-FU interferes with nucleoside metabolism and can be incorporated into RNA and DNA, leading to cytotoxicity and cell death [5,6].

Over the past 50 years, despite its many advantages, clinical applications have been greatly limited due to drug resistance. The overall response rate for advanced colorectal cancer of 5-FU alone is still only 10–15% [7], and the combination of 5-FU with other anti-tumor drugs has merely improved the response rates to 40–50% [8]. Therefore, new strategies for therapy and resistance reversal are urgently needed. Meanwhile, understanding the mechanisms by which tumors become resistant to 5-FU is an essential step towards predicting or overcoming that resistance. Fortunately, the development of microarray techniques offers us a chance to identify new genes which have key roles in drug resistance. Now, we can move forward to investigate the mechanism of these molecules, which might contribute to clinical chemotherapy in the future.

## Mechanism of action

In mammalian cells, 5-FU is converted to fluorodeoxyuridine monophosphate (FdUMP), which forms a stable complex with thymidylate synthase (TS), and thus inhibits deoxythymidine mono-phosphate (dTMP) production. dTMP is essential for DNA replication and repair and its depletion therefore causes cytotoxicity [9,10]. Dihydropyrimidine dehydrogenase (DPD)-mediated conversion of 5-FU to dihydrofluorouracil (DHFU) is the rate-limiting step of 5-FU catabolism in normal and tumor cells. Up to 80% of administered 5-FU is broken down by DPD in the liver [11].

### TS inhibition

TS, an essential enzyme for catalyzing the biosynthesis of thymidylate, is implicated in the regulation of protein synthesis and apoptotic processes [12,13]. TS catalyzes the methylation of deoxyuridine monophosphate (dUMP) to dTMP, for which 5,10-methylenetetrahydrofolate (CH_2_THF) is the methyl donor, and finally provides with the reaction thymidylate to maintain DNA replication and repair [14]. The reaction has a seriatim binding sequence, and dUMP binds at the active site before CH_2_THF does. Then the reaction is initiated by the nucleophilic addition of the active site Cys 146 (numbering of amino acid residues used is according to the sequence of EcTS) to the pyrimidine C (6)atom of dUMP. Specifically, at the onset of catalysis, the binding position and orientation of the substrate, if correctly adopted, support an efficient binding of the cofactor, and then allow the formation of the ternary TS–dUMP–CH_2_THF complex, and the subsequent reaction [15].

Research has indicated that 5-FU exerts its anticancer effects mainly through inhibition of TS, for which the pathways have not been fully interpreted. Santi has pointed out that the formation of the ternary TS–FdUMP–CH_2_THF complex is time-dependent, and the reaction stops as the ﬂuorine substituent fails to dissociate from the pyrimidine ring, resulting in a slowly reversible inactivation of the enzyme [16]. Reduction of dTMP leads to downstream depletion of deoxythymidine triphosphate (dTTP), which induces perturbations in the levels of the other deoxynucleotides (dATP, dGTP and dCTP). Finally the imbalances (the ATP/dTTP ratio specifically) are thought to severely disrupt DNA synthesis and repair, resulting in lethal DNA damage [17,18]. Accumulation of dUMP, which might subsequently lead to increased levels of deoxyuri-dine triphosphate (dUTP), can be misincorporated into DNA, and FdUTP, the metabolic product of 5-FU has the same action [19]. Furthermore, repairing enzyme uracil-DNA-glycosy-lase (UDG) is suggested to be useless in the presence of high (F) dUTP/dTTP ratios and only results in further false DNA repair [20].

### DNA and RNA misincorporation

5-FU is a pyrimidine analogue that can be misincorporated into RNA and DNA in place of uracil or thymine. The interference with the normal biosynthesis or function of nucleic acids is therefore another possible mechanism of action for 5-FU. 5-FU can be misincorporated into DNA of drug-treated cells, and accumulation of 5-FU in the genome, rather than uracil excision, is correlated with 5-FU cytotoxicity in mammalian cells [21]. It can also be misincorporated into RNA, and evidence suggests that RNA-based effects play a significant role in its cytotoxicity. Experiments in yeast show that defects in the nuclear RNA exosome subunit Rrp6p could cause hypersensitivity to 5-FU. Genetic analyses suggest that while a DNA repair mutation, apn1-Δ causes sensitivity to 5-FU-induced DNA damage, and an rrp6-Δ mutation causes hypersensitivity, due to the RNA-based effects of 5-FU [22].

The results suggest that rRNA maturation is an important target for 5-FU [23,24,25]. 5-FU has also been shown to enhance exosome-dependent accumulation of polyadenylated rRNAs [26]. And there is evidence that 5-FU inhibits premRNA splicing, through its effect on pseudouridylation of U2 snRNA [27].

The action of 5-FU is not only inhibiting the processing of pre-rRNA into mature rRNA, but also disrupting post-transcriptional modification of tRNAs and the assembly activity of snRNA/protein complexes, thereby inhibiting splicing of pre-mRNA [28]. 5-FU-containing RNA can also inhibit pseudouridylation, the most abundant post-transcriptional modification of noncoding RNA. Results suggest that Cbf5p binds tightly to substrates containing 5-FU, causing their degradation by the TRAMP/exosome-mediated RNA surveillance pathway. And the RNA-based 5-FU toxicity requires the pseudouridylation activity of Cbf5p, and sequestration of Cbf5p to a particular guide RNA reduced Cbf5p-dependent 5-FU toxicity [29].

**Figure 1:**
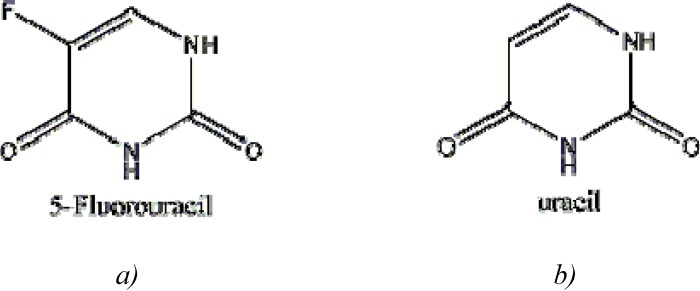
a) 5-FU, b) uracil.

### Assisted modulation

### Leucovorin

Leucovorin (LV), which enters the cell via the reduced folate carriers and is anabolized to CH_2_THF, increases the intracellular CH_2_THF pool, thereby enhancing TS inhibition by FdUMP [30]. During the procedure, CH_2_THF is polyglutamated by folylpolyglutamate synthetase. Polyglutamation not only increases the cellular retention of CH_2_THF, but also enhances the stabilization of its ternary complex with TS and FdUMP [31,32]. However, resistance to modulation of 5-FU by LV exists and decreased stability of ternary complexes appears to be the mechanism of acquired resistance to the LV modulation of fluoropyrimidine cytotoxicity, possibly due to mutation(s) of TS [33,34,35].

### Interferons

Interferons (IFNs) are pleiotropic cytokines that exert negative regulatory effects on the growth of normal and malignant cells *in vitro* and *in vivo*. Studies have reported that IFNs might enhance the cytotoxicity of 5-FU in various cancer cell lines. 5-FU might induce secretion of proteolytically processed mature and degraded IL-18 species, by inducing Caspase-1 and Caspase-3 activation. Conditioned medium from 5-FU-treated induces IFN-g production by activated T cells in an IL-18-dependent manner. Carbone *et al.* [36] suggest treatment of pancreatic cancer cells with 5-FU induces caspase-dependent processing of pro-IL18 leading to the secretion of biologically active IL-18. Results show that combination of IFN-A plus 5-FU strongly induces cell growth inhibition of human hepatocellular carcinoma cells and indicates that one of the direct mechanisms of combination therapy may in part be attributable to alterations in induction of apoptosis through IFN-A/BR [37]. Wada *et al*. [38] suggest IFN-alpha and 5-FU combination therapy has anti-proliferative and anti-angiogenic effects and can induce apoptosis *in vivo*.

### Apoptosis

Enhancing the sensitization of cancer cells to drug-induced apoptosis has become an important strategy for 5-FU. 5-FU might trigger the cancer cell apoptosis by activating caspase-6. RSV synergistically promotes 5-FU-mediated apoptosis at its higher concentration irrespective of p53 [39]. The 5-FU also generates mitochondrial ROS in the p53-dependent pathway [40]. In addition, BMI-1 depletion enhances the chemosensitivity of NPC cells by inducing apoptosis; which is associated with inhibition of the PI3K/AKT pathway [41].

### Cell cycle

Results suggest that 5-FU could induce changes in cell cycle regulation, which might associate with an alteration of G1 cyclins expression [42]. The *in vitro* 5-FU treatment of oral cancer cells results in an increase in G1/S phase cells, and p21 is remarkably up-regulated. A remarkable up-regulation of cyclin E and a concomitant down-regulation of cyclin D are observed. There is also speculation that its activity might be cancelled by increased binding to CDK4.

### L-Arginine

5-FU combined with L-arginine (L-Arg) could inhibit the growth of tumor in nude mice. The effect may be related to inducing the synthesis and increasing the activity of endogenous nitric oxide (NOS). The production of NO is increased, and it can enhance the expression of apoptosis-related gene and antioncogene [43].

### Others

Ooyama *et al*. [45] suggested that 5-FU could also induce cytotoxic activity by inhibition of angiogenesis through the induction of thrombospondin-1 (TSP-1). Hwang *et al.* [46] proposed that the combination of 5-FU and genistein exert a novel chemotherapeutic effect on colon cancers, and AMPK might be a novel regulatory molecule of COX-2 expression, further implying its involvement in cytotoxicity caused by genistein.

## Resistance

Anti-cancer drug resistance can result from various causes including alteration of drug influx and efflux, enhancement of drug inactivation and mutation of the drug target [47]. High-level expression of TS [48], increased activity of deoxyuridine triphosphatase [49], methylation of the MLH1 gene [50], and overexpression of Bcl-2 [51], Bcl-XL [51,52], and Mcl-1 [53] proteins have all been reported to lead to resistance to 5-FU, which suggests that multiple factors might contribute to 5-FU resistance [54,55].

### TS

There are several possible aspects for TS induction: decreased accumulation of activated metabolites, target-associated resistance and pharmacokinetic resistance [56]. The stability of the ternary complex is highly dependent on the availability of CH_2_THF or one of its polyglutamates [57,58]. Thymidylate can be salvaged from thymidine through the action of thymidine kinase, thereby alleviating the effects of TS deficiency. This salvage pathway represents a potential mechanism of resistance to 5-FU [59]. The binding of ligands to the TS molecule leads to dramatic changes in the conformation of the enzyme, particularly within the C-terminal domain. Stabilization of the enzyme and an increase in its intracellular level are associated with ligand binding and may be important in cellular response to TS-directed drugs. The C-terminal conformational shift is not required for ligand-mediated stabilization of the enzyme TS, while the N-terminus of the TS polypeptide, which is extended in the mammalian enzyme and is disordered in crystal structures, is a primary determinant of the enzyme's half-life, and TS turnover is carried out by the 26S proteasome in a ubiquitin-independent manner [60,61,62]. Further, the penultimate amino acid Pro2, which is capable on its own of destabilizing an evolutionarily distinct TS molecule, plays an important role in governing the half-life of the enzyme [63].

TS over-expression is widely accepted as a major molecular mechanism responsible for 5-FU resistance. The stability of the ternary complex is highly dependent on the availability of CH_2_THF, and in the absence of CH_2_THF or one of its polyglutamates, FdUMP forms an unstable binary complex, which results in poor inhibition [64,65]. Disturbed folate pools and a high level of enzyme before treatment lead to intrinsic resistance [66,67]. Gene amplification of TS and mutations in the gene lead to acquired resistance [67,68]. In addition, thymidylate can be salvaged from thymidine through the action of thymidine kinase, thereby alleviating the effects of TS deficiency. This salvage pathway represents a potential mechanism of resistance to 5-FU [69]. The acute induction in TS levels following therapy with inhibitors of this enzyme is also one of the critical mechanisms of resistance to 5-FU. This mechanism is based on a novel autoregulatory feedback pathway wherein the TS protein regulates its own translational efficiency [70]. Wang *et al.* have elucidated that TS-independent molecular events might play a key role in 5-FU resistance [71].

### DPD

Increased DPD activity and the corresponding catabolism of 5-FU may lead to 5-FU resistance. Studies show that low-DPD tumors could be sensitive to 5-FU [44], and Danenberg *et al.* have revealed that DPD mRNA expression in colorectal metastatic or disseminated tumors is related to the anti-tumor effect of 5-FU [112]. At least 39 different alleles in the DPYD gene encoding DPD protein have been identified, and a potential prominent mutation affecting the splicing donor consensus sequence of intron 14 has been reported, which results in the deletion of 55 amino acids in the native protein [113]. However, no clear correlation between 5-FU-associated toxicity phenotype and genotype has been established thus far [11].

### DNA and RNA misincorporation

Tajima *et al*. [72] suggested that MMR complex hMutS alpha might be specifically recognized and bound to 5-FU-modified DNA. Evidence suggests that tumor cells with MSI (caused by defective DNA mismatch repair) are more resistant to 5-FU in culture compared with microsatellite stable cells, despite similar amounts of 5-FU misincorporation into the cell's DNA. The reaction is specific as added ATP dissociates the hMutS alpha complex from the 5-FU-modified strand. And there was greater binding between hMutS alpha and 5-FU-modified DNA compared with complementary DNA or DNA containing a C/T mismatch. Qian An *et al*. [21] suggested that Smug1, but not UNG (uracil-DNA glycosylase), could excise 5-FU from DNA and protect against cell killing, as a predictive biomarker of drug response and a mechanism for acquired resistance in tumors.

### Anti-apoptosis

The oncogene B-cell-specific Moloney murine leukemia virus insertion site 1 (BMI-1) has been shown to be involved in the protection of cancer cells from apoptosis. The expression of phospho-AKT and the anti-apoptotic protein BCL-2 are downregulated in the cells in which BMI-1 expression is inhibited, whereas the apoptosis-inducer BAX is observed to be upregulated. Abrogation of AKT pathway by a PI3K inhibitor could not further increase the sensitivity to 5-FU in the cells with reduced BMI-1 expression [41].

Human Ring-Finger, homologous to Inhibitor of Apoptosis protein type (hRFI) has been shown to inhibit death receptor mediated apoptosis. Evidence suggests that the modulation of Bcl-2 family proteins seen in 5-FU treatment plays an important role in the anti-apoptotic function. This might also clarify hRFI overexpression and the manner in which hRFI upregulates Bcl-2 and Bcl-XL and elevates the relative ratio of Bcl-2 to Bax or to Bak during 5-FU treatment [73]. Furthermore, hRFI overexpression results in the activation of nuclear factor-jB (NF-jB). Inhibition of NF-jB might effectively reverse the resistance to apoptosis as well as the upregulation of Bcl-2 and Bcl-XL in the hRFI transfectant, indicating that the activation of NF-jB is the key mechanism [74].

Tseng *et al*. [75] suggest that Ras, Bcl-2, as well as Raf-1 and PI3K pathways play pivotal roles in 5-FU-induced apoptosis under Ha-ras-overexpressed condition. Aberrant levels of cyclin E and p21Cip/WAF-1 expression, as well as Cdc 2 phosphorylation at Tyrosine 15, suggest that perturbation of G1/S and G2/M transitions in cell cycle might be responsible for 5-FU triggered apoptosis. Besides, the resistance could be related to an increase in the expression of IAP survivin, which can decrease cell response to the treatment or even switch the type of death from apoptosis to another kind, making therapy less efficient [76].

### Cell cycle

Cell cycle perturbation may be involved in acquired 5-FU resistance. There might be a slow down in cell cycle traverse preventing incorporation of 5-FU metabolites into DNA, providing cancer cells with sufficient time to correct the misincorporated nucleotides. The resistant cell lines show significantly lower labelling indexes and cell cycle delays in G1 and G1/S boundary and prolonged DNA synthesis time. Meanwhile, the resistant cell lines demonstrate significantly prolonged potential doubling time (Tpot). CDK2 protein, Thr-160 phosphorylated CDK2, cyclin D3 and cyclin A might be remarkably reduced in the resistant cell lines [77].

### NO

5-FU is shown to inhibit NO production, indicating that 5-FU inhibits high levels of NO output in activated macrophages. High levels of NO generated by activated macrophages play an important role on anti-tumor activity in tumor immunity [78]. Therefore, 5-FU is very possible to attenuate anti-tumor activity of activated macrophages.

5-FU might prevent LPS-induced NO production via inactivation of the Akt-dependent NF-κB signal pathway. The possibility that 5-FU inhibits the NO production via its cytotoxic action is excluded. The studies show that 5-FU inhibits the expression of iNOS mRNA and protein, such as signal transduction in LPS stimulation. Moreover, 5-FU might inhibit the phosphorylation of Akt that regulates NF-κB activation as an upstream molecule through IKK activation [79], and the failure of NF-κB activation results in attenuated expression of iNOS protein and subsequent NO production [80].

### Mitochondria

ATP synthase down-regulation may lead to cellular events responsible for 5-FU resistance. The studies show there is lower expression of the A subunit of mitochondrial F1F0-ATP synthase (ATP synthase) and other ATP synthase complex subunits in 5-FU–resistant cells. Thus ATP synthase activity is decreased and intracellular ATP content reduced. The ATP synthase inhibitor oligomycin A strongly antagonizes 5-FU–induced suppression of cell proliferation [81].

### Oxidative stress

Cellular adaptive response to ROS is another mechanism of drug resistance to 5-FU. While acute oxidative stress triggers cell apoptosis or necrosis, persistent oxidative stress induces genomic instability and has been implicated in tumor progression and drug resistance. Studies show that tumor cells which adapt to oxidative stress by increasing manganese superoxide dismutase (MnSOD), Prx I and Bcl-2, show drug resistance to 5-FU. Romo1 siRNA treatment efficiently blocks 5-FU-induced ROS generation, demonstrating that 5-FU treatment stimulates ROS production through Romo1 induction [82].

### Interferons

Induction of TSt might represent a relevant mechanism of resistance to 5-FU and this mechanism can be circumvented in the presence of IFNγ. Studies suggest IFNγ is potent in prolonging the antitumor effect of 5-FU by suppressing an acute overexpression of TSt. The triple combination therapy and combination therapy (5-FU and IFNγ) show significant growth suppression of cancer cells when compared with 5-FU [83,84,85].

### Other molecules

The results indicate regulation of midkine gene expression in cancer cells (MDK) appears to modulate sensitivities to anticancer drugs. *De novo* expression of MDK in cancer cells not normally expressing MDK confers a multi-drug resistance, while knockdown of MDK in cancer cells that normally expressed the protein leads to chemosensitization [86]. In addition, Chu *et al*. [87] suggest that increased metabotropic glutamate receptor 4 (mGluR4) expression is a mechanism underlying 5-FU resistance. The ATP-binding cassette (ABC) proteins play an important role in drug resistance of 5-FU, and ABCC5 is one of the multidrug resistance (MDR) subfamily of ABC proteins [88,89]. Fanciullino *et al*. [90] propose that nuclear expression of rTS beta could be a novel 5-FU resistance marker in patients with primary breast cancer. And results suggest that hENT1 plays an important role in 5-FU resistance and that hENT1 mRNA levels might be a useful marker to predict 5-FU sensitivity in pancreatic cancer [91].

## Gene arrays

Microarray technology, developed in recent years, has enabled analysis of pan-genomic expression profiles in cells or tissues of interest. A combination of microarray and traditional molecular technologies will enable us to functionally characterize genes related to anticancer drug resistance and identify novel molecular targets for anticancer drug development.

Genetic factors might play an important role in resistance towards 5-FU, and genetic variation in any of these may contribute to anti-tumor response. Gene expression data suggest that altered regulation of nucleotide metabolism, amino acid metabolism, cytoskeleton organization, transport, and oxygen metabolism may underlie the differential resistance to 5-FU seen in cell lines.

We have obtained a number of genes directly associated with sensitivity or resistance to 5-FU identified by micro-array techniques. These results provide not only predictive biomarkers for 5-FU sensitivity or resistance to human cancers, but also a new molecular basis for understanding the mechanism of cellular cytotoxicity to 5-FU [92,93]. Differential expressions in response to 5-FU treatment are demonstrated for genes involved in regulation of nucleotide binding/metabolism (ATAD2, GNL2, GNL3, MATR3), amino acid metabolism (AHCY, GSS, IVD, OAT), cytoskeleton organization (KRT7, KRT8, KRT19, MAST1), transport (MTCH1, NCBP1, SNAPAP, VPS52), oxygen metabolism (COX5A, COX7C) [94,95], metastasis (LMNB1, F3, TMSNB), apoptosis-promoting (BNIP3, BNIP3L, FOXO3A), positive growth-regulatory (CCND3, CCNE2, CCNF, CYR61), negative growth-regulatory (AREG, CCNG2, CDKN1A, CDKN1C, GADD45A), and DNA repair (FEN1, FANCG, RAD23B) [96]. And among the up-regulated genes, two genes (PTGS2 and CLU) are particularly of interest [86].

Several highly significant associations have been observed between genotypes and expression levels of 5-FU metabolizing genes. In a study, Nordgard *et al*. [97] found a SNP in codon 72 of TP53 is a key regulator of 5-FU metabolizing genes such as DHFR and MTHFR. These data suggest that three copies of the TYMS 50UTR repeat might give a treatment specific reduced survival in breast cancer patients, and that TP53 might have a direct, allele specific, role in 5-FU mediated response. Another analysis confirms that the SNP showing significant associations with drug sensitivity are concentrated in some cytogenetic regions (18p, 17p13.2, 17p12, 11q14.1, 11q11 and 11p11.12). Among these regions, 18p11.32 at the location of the thymidylate synthase gene (TYMS) is strongly associated with resistance to 5-FU-based drugs. The results suggest that amplification of the TYMS gene is associated with innate resistance, supporting the possibility that TYMS copy number might be a predictive marker of drug sensitivity to fluoropyrimidines [98].

Different stages of FU resistance of low-, intermediate- and high-resistance phenotypes are also studied [99]. Some research has also used statistics tools, like the Java program TOUCAN, to identify a consensus gene list associated with 5-FU resistance, perform an *in silico* comparative promoter analysis, and highlight the potential implication of some TFs in the development of chemoresistance [100].

## Future perspectives

Although 5-FU and its derivatives [101,102] have been important anticancer agents and have been widely applied in patients, the overall response rate of 5-FU alone is still low. Therefore, seeking the better therapeutic strategies, increase 5-FU sensitivity and reverse the resistance to drug are the key tasks in the future.

### Therapeutic strategies

Studies suggest that resistance to 5-FU could be overcome through a better control of its intratumoural activation and the use of an encapsulated formulation [90]. As drug deliverers, liposomes could congregate drugs in certain tissues, decrease poisonous effects and increase the curative effects. Lecithoid material is the most primary liposome commonly used. Since Glycoprotein C (GC) is a cerebroside containing galactose, liposome made of GC also possesses galactose. Compared with lecithoid, GC has several advantages such as stable chemical characteristics, long retention in blood, anti-oxide ability and unique directional trait. It may be developed into a new kind of medicine carriers.

Combination chemotherapy regimens, including FOLFIRI and FOLFOX, have prolonged the survival of advanced or metastatic cancer patients compared with BSC alone. Additional monoclonal antibody agents provide little additive utility at high cost. New agents, including new macromolecule agents, small molecule agents and vaccines, will be introduced in the chemotherapy against colorectal cancer. Subsequently, clinical researchers will have to consider the cost-utility of these agents using QALY [35].

### Reversal of resistance

Some of the enzymes involved in the metabolic process of 5-FU, including thymidylate synthase and dihydropyrimidine dehydrogenase, have been shown to predict sensitivity to 5-FU and/or prognosis [103,104]. Based on the fact that the inhibitor of DPD has been shown to enhance the anticancer effects of 5-FU [105], DPD-inhibitory fluoropyrimidines (DIF) have been developed to enhance anticancer effects. To date, DIFs have played a major role in neoadjuvant and/or adjuvant chemotherapy for patients with advanced gastric carcinoma [106]. And the measurement of orotate phosphoribosyltransferase (OPRT) in cancer tissue may be useful for the prediction and monitoring of the anticancer effects of S-1-based anticancer chemotherapy for patients with advanced cancers [107].

YSV could effectively reverse MDR associated with the down-regulation of MDR1, MRP1 and LRP expression, as well as the inhibition of P-gp function [108]. Studies also show that the Smug1, but not the UNG, excises FU from DNA and protects against cell killing [21]. Wild-type p53 gene has a remarkable reversal activity for the high expression of MDR1 gene in colorectal cancers. The reversal effects seem to be in a time dependent manner [109]. Zhu *et al.* [110] suggest knockdown of Bcl-XL protein levels by small interfering RNA (siRNA) inhibits the proliferation more effectively in 5-FU-resistant cells than in 5-FU-sensitive cells, and down-regulation of Bcl-XL protein expression might provide a new treatment strategy for human 5-FU-resistant colon cancer therapy. Besides, they also [111] suggest that colon cancer cells resistant to tumor necrosis factor–related apoptosis-inducing ligand (TRAIL) could be resensitized by a combination therapy of TRAIL plus 5-FU.

## Conclusions

As described above, there are two main anti-tumor mechanisms of 5-FU which have been proven. 5-FU is an effective chemotherapeutic drug developed as an inhibitor of TS, which leads to a thymine-less cell death. And it is also a pyrimidine analogue misincorporated into RNA and DNA in place of uracil or thymine. However, its clinical application is greatly limited due to drug resistance, which could result from various causes, including alteration of drug influx and efflux, enhancement of drug inactivation and mutations of the drug target. Surely there could be still many mechanisms of 5-FU anti-tumor action and drug resistance, which have not been demonstrated yet. The new technologies, such as microarrays, might enable us to functionally characterize genes related to these mechanisms more effectually.

## References

[1] Grem J.L. (2000). 5-Fluorouracil: forty-plus and still ticking. A review of its preclinical and clinical development. Invest New Drugs.

[2] Rutman R.J., Cantarow A., Paschkis K.E. (1954). Studies on 2-acetylaminofluorene carcinogenesis: III. The utilization of uracil-2-C14 by pre–neoplastic rat liver. Cancer Res..

[3] Hulme A.T., Price S.L., Tocher D.A. (2005). A New Polymorph of 5-Fluorouracil Found Following Computational Crystal Structure Predictions. J. Am. Chem. Soc..

[4] Singh U.P., Ghose R., Ghose A.K., Sodhi A., Singh S.M., Singh R.K. (1989). The effect of histidine on the structure and antitumor activity of metal-5-halouracil complexes. J. Inorg. Biochem..

[5] Thomas D.M., Zalcberg J.R. (1998). 5-fluorouracil: a pharmacological paradigm in the use of cytotoxics. Clin. Exp. Pharmacol. Physiol..

[6] Noordhuis P., Holwerda U., Van der Wilt C.L., Van Groeningen C.J., Smid K., Meijer S., Pinedo H.M., Peters G.J. (2004). 5-Fluorouracil incorporation into RNA and DNA in relation to thymidylate synthase inhibition of human colorectal cancers. Ann. Oncol..

[7] Giacchetti S., Perpoint B., Zidani R., Le Bail N., Faggiuolo R., Focan C., Chollet P., Llory J.F., Letourneau Y., Coudert B., Bertheaut-Cvitkovic F., Larregain-Fournier D., Le Rol A., Walter S., Adam R., Misset J.L., Lévi F. (2000). Phase III multicenter randomized trial of oxaliplatin added to chronomodulated fluorouracilleucovorin as first-line treatment of metastatic colorectal cancer. J. Clin. Oncol..

[8] Douillard J.Y., Cunningham D., Roth A.D., Navarro M., James R.D., Karasek P., Jandik P., Iveson T., Carmichael J., Alakl M., Gruia G., Awad L., Rougier P. (2000). Irinotecan combined with fluorouracil compared with fluorouracil alone as first-line treatment for metastatic colorectal cancer: a multicentre randomised trial. Lancet.

[9] Parker W.B., Cheng Y.C. (1990). Metabolism and mechanism of action of 5-fluorouracil. Pharmacol. Ther..

[10] Longley D.B., Latif T., Boyer J., Allen W.L., Maxwell P.J., Johnston P.G. (2003). The interaction of thymidylate synthase expression with p53-regulated signaling pathways in tumor cells. Semin. Oncol..

[11] He Y.F., Wei W., Zhang X., Li Y.H., Li S., Wang F.H., Lin X.B., Li Z.M., Zhang D.S., Huang H.Q., Hu B., Jiang W.Q. (2008). Analysis of the DPYD gene implicated in 5-fluorouracil catabolism in Chinese cancer patients. J. Clin. Pharm. Ther..

[12] Bruni P., Minopoli G., Brancaccio T., Napolitano M., Faraonio R., Zambrano N., Hansen U., Russo T. (2002). Fe65, a ligand of the Alzheimer's beta-amyloid precursor protein, blocks cell cycle progression by down-regulating thymidylate synthase expression. J. Biol. Chem..

[13] Chernyshev A., Fleischmann T., Kohen A. (2007). Thymidyl biosynthesis enzymes as antibiotic targets. Appl. Microbiol. Biotechnol..

[14] Roberts S.A., Hyatt D.C., Honts J.E., Changchien L., Maley G.F., Maley F., Montfort W.R. (2006). Structure of the Y94F mutant of Escherichia coli thymidylate synthase. Acta Crystallogr. Sect. F. Struct. Biol. Cryst Commun..

[15] Newby Z., Lee T.T., Morse R.J., Liu Y., Liu L., Venkatraman P., Santi D.V., Finer-Moore J.S., Stroud R.M. (2006). The role of protein dynamics in thymidylate synthase catalysis: variants of conserved 2'-deoxyuridine 5'-monophosphate (dUMP)-binding Tyr-261. Biochemistry.

[16] Sotelo-Mundo R.R., Changchien L., Maley F., Montfort W.R. (2006). Crystal structures of thymidylate synthase mutant R166Q: structural basis for the nearly complete loss of catalytic activity. J. Biochem. Mol. Toxicol..

[17] Danenberg P.V. (1977). Thymidylate synthetase - a target enzyme in cancer chemotherapy. Biochim. Biophys. Acta..

[18] Jarmuła A., Dowierciał A., Rode W. (2008). A molecular modeling study of the interaction of 2'-fluoro-substituted analogues of dUMP/FdUMP with thymidylate synthase. Bioorg. Med. Chem. Lett..

[19] Santi D.V., McHenry C.S., Raines R.T., Ivanetich K.M. (1987). Kinetics and thermodynamics of the interaction of 5-fluoro-2'-deoxyuridylate with thymidylate synthase. Biochemistry.

[20] Houghton J.A., Tillman D.M., Harwood F.G. (1995). Ratio of 2'-deoxyadenosine-5'-triphosphate/thymidine-5'-triphosphate influences the commitment of human colon carcinoma cells to thymineless death. Clin. Cancer Res..

[21] An Q., Robins P., Lindahl T., Barnes D.E. (2007). 5-Fluorouracil incorporated into DNA is excised by the Smug1 DNA glycosylase to reduce drug cytotoxicity. Cancer Res..

[22] Hoskins J., Scott Butler J. (2007). Evidence for distinct DNA- and RNA-based mechanisms of 5-fluorouracil cytotoxicity in Saccharomyces cerevisiae. Yeast.

[23] Gustavsson M., Ronne H. (2008). Evidence that tRNA modifying enzymes are important in vivo targets for 5-fluorouracil in yeast. RNA.

[24] Giaever G., Flaherty P., Kumm J., Proctor M., Nislow C., Jaramillo D.F., Chu A.M., Jordan M.I., Arkin A.P., Davis R.W. (2004). Chemogenomic profiling: identifying the functional interactions of small molecules in yeast. Proc. Natl. Acad. Sci. U S A.

[25] Lum P.Y., Armour C.D., Stepaniants S.B., Cavet G., Wolf M.K., Butler J.S., Hinshaw J.C., Garnier P., Prestwich G.D., Leonardson A., Garrett-Engele P., Rush C.M., Bard M., Schimmack G., Phillips J.W., Roberts C.J., Shoemaker D.D. (2004). Discovering modes of action for therapeutic compounds using a genome-wide screen of yeast heterozygotes. Cell.

[26] Fang F., Hoskins J., Butler J.S. (2004). 5-fluorouracil enhances exosome-dependent accumulation of polyadenylated rRNAs. Mol. Cell Biol..

[27] Zhao X., Yu Y.T. (2007). Incorporation of 5-fluorouracil into U2 snRNA blocks pseudouridylation and pre-mRNA splicing in vivo. Nucleic Acids Res..

[28] Samuelsson T. (1991). Interactions of transfer RNA pseudouridine synthases with RNAs substituted with fluorouracil. Nucleic Acids Res..

[29] Hoskins J., Butler J.S. (2008). RNA-Based 5-Fluorouracil Toxicity Requires the Pseudouridylation Activity of Cbf5p. Genetics.

[30] Longley D.B., Harkin D.P., Johnston P.G. (2003). 5-fluorouracil: mechanisms of action and clinical strategies. Nat. Rev. Cancer.

[31] Dolnick B.J., Cheng Y.C. (1978). Human thymidylate synthetase. II. Derivatives of pteroylmono- and -polyglutamates as substrates and inhibitors. J. Biol. Chem..

[32] Radparvar S., Houghton P.J., Houghton J.A. (1989). Effect of polyglutamylation of 5,10-methylene-tetrahydrofolate on the binding of 5-fluoro-2′-deoxyuridylate to thymidylate synthase purified from a human colon adenocarcinoma xenograft. Biochem. Pharmacol..

[33] Lu K., McGuire J.J., Slocum H.K., Rustum Y.M. (1997). Mechanisms of acquired resistance to modulation of 5-fluorouracil by leucovorin in HCT-8 human ileocecal carcinoma cells. Biochem. Pharmacol..

[34] Spears C.P., Gustavsson B.G., Berne M., Frösing R., Bernstein L., Hayes A.A. (1988). Mechanisms of innate resistance to thymidylate synthase inhibition after 5-fluorouracil. Cancer Res..

[35] Omura K. (2008). Advances in Chemotherapy against Advanced or Metastatic Colorectal Cancer. Digestion.

[36] Carbone A., Rodeck U., Mauri F.A., Sozzi M., Gaspari F., Smirne C., Prati A., Addeo A., Novarino A., Robecchi A., Bertetto O., Emanuelli G., Bellone G. (2005). Human Pancreatic Carcinoma Cells Secrete Bioactive Interleukin-18 after Treatment with 5-Fluorouracil. Cancer Biol. Ther..

[37] Kondo M., Nagano H., Wada H., Damdinsuren B., Yamamoto H., Hiraoka N., Eguchi H., Miyamoto A., Yamamoto T., Ota H., Nakamura M., Marubashi S., Dono K., Umeshita K., Nakamori S., Sakon M., Monden M. (2005). Combination of IFN-A and 5-Fluorouracil Induces Apoptosis through IFN-A/B Receptor in Human Hepatocellular Carcinoma. Clin. Cancer Res..

[38] Wada H., Nagano H., Yamamoto H., Arai I., Ota H., Nakamura M., Damdinsuren B., Noda T., Marubashi S., Miyamoto A., Takeda Y., Umeshita K., Doki Y., Dono K., Nakamori S., Sakon M., Monden M. (2007). Combination therapy of interferon-alpha and 5-fluorouracil inhibits tumor angiogenesis in human hepatocellular carcinoma cells by regulating vascular endothelial growth factor and angiopoietins. Oncol. Rep..

[39] Chan J.Y., Phoo M.S., Clement M.V., Pervaiz S, Lee S.C. (2008). Resveratrol displays converse dose-related effects on 5-fluorouracil-evoked colon cancer cell apoptosis: the roles of caspase-6 and p53. Cancer Biol. Ther..

[40] Hwang P.M., Bunz F., Yu J., Rago C., Chan T.A., Murphy M.P., Kelso G.F., Smith R.A., Kinzler K.W, Vogelstein B. (2001). Ferredoxin reductase affects p53-dependent, 5-fluorouracil-induced apoptosis in colorectal cancer cells. Nat. Med..

[41] Qin L., Zhang X., Zhang L., Feng Y., Weng G.X., Li M.Z., Kong Q.L., Qian C.N., Zeng Y.X., Zeng M.S., Liao D.F., Song L.B. (2008). Downregulation of BMI-1 enhances 5-fluorouracil-induced apoptosis in nasopharyngeal carcinoma cells. Biochem. Biophys. Res. Commun..

[42] Li M.H., Ito D., Sanada M., Odani T., Hatori M., Iwase M., Nagumo M. (2004). Effect of 5-fluorouracil on G1 phase cell cycle regulation in oral cancer cell lines. Oral Oncol..

[43] Yin X.Y., Jiang J.M., Liu J.Y., Zhu J.R. (2007). Effects of endogenous nitric oxide induced by 5-fluorouracil and L-Arg on liver carcinoma in nude mice. World J. Gastroenterol..

[44] Fukuda H., Takiguchi N., Koda K., Oda K., Seike K., Miyazaki M. (2006). Thymidylate Synthase and Dihydropyrimidine Dehydrogenase are related to histological effects of 5-Fluorouracil and cisplatin neoadjuvant chemotherapy for primary gastric cancer patients. Cancer Invest..

[45] Ooyama A., Oka T., Zhao H.Y., Yamamoto M., Akiyama S.I., Fukushima M.  (2008). Anti-angiogenic effect of 5-Fluorouracil-based drugs against human colon cancer xenografts. Cancer Lett..

[46] Hwang J.T., Ha J., Park O.J. (2005). Combination of 5-Fluorouracil and genistein induces apoptosis synergistically in chemo-resistant cancer cells through the modulation of AMPK and COX-2 signaling pathways. Biochem. Biophys. Res. Commun..

[47] Longley D.B., Johnston P.G. (2005). Molecular mechanisms of drug resistance. J. Pathol..

[48] Van Triest B., Pinedo H.M., Van Hensbergen Y., Smid K., Telleman F., Schoenmakers P.S., Van der Wilt C.L., Van Laar J.A., Noordhuis P., Jansen G., Peters G.J. (1999). Thymidylate synthase level as the main predictive parameter for sensitivity to 5-fluorouracil, but not for folate-based thymidylate synthase inhibitors, in 13 nonselected colon cancer cell lines. Clin. Cancer Res..

[49] Grem J.L. (2005). Screening for dihydropyrimidine dehydrogenase deficiency. Clin. Cancer Res..

[50] Arnold C.N., Goel A., Boland C.R. (2003). Role of hMLH1 promoter hypermethylation in drug resistance to 5-fluorouracil in colorectal cancer cell lines. Int. J. Cancer.

[51] Violette S., Poulain L., Dussaulx E., Pepin D., Faussat A.M., Chambaz J., Lacorte J.M., Staedel C., Lesuffleur T. (2002). Resistance of colon cancer cells to long-term 5-fluorouracil exposure is correlated to the relative level of Bcl-2 and Bcl-X(L) in addition to Bax and p53 status. Int. J. Cancer.

[52] Liu R., Page C., Beidler D.R., Wicha M.S., Núñez G. (1999). Overexpression of Bclx(L) promotes chemotherapy resistance of mammary tumors in a syngeneic mouse model. Am. J. Pathol..

[53] Shi X., Liu S., Kleeff J., Friess H., Büchler M.W. (2002). Acquired resistance of pancreatic cancer cells towards 5-Fluorouracil and gemcitabine is associated with altered expression of apoptosis-regulating genes. Oncology.

[54] Miyashita T. (1994). Tumor suppressor p53 is a regulator of Bcl-2 and Bax gene expression in vitro and in vivo. Oncogene.

[55] Petak I., Tillman D.M., Houghton J.A. (2000). p53 dependence of Fas induction and acute apoptosis in response to 5-fluorouracil-leucovorin in human colon carcinoma cell lines. Cancer Res..

[56] Yoshioka A., Tanaka S., Hiraoka O., Koyama Y., Hirota Y., Ayusawa D., Seno T., Garrett C., Wataya Y. (1987). Deoxyribonucleoside triphosphate imbalance. 5-Fluorodeoxyuridine-induced DNA double strand breaks in mouse FM3A cells and the mechanism of cell death. J. Biol. Chem..

[57] Aherne G.W., Hardcastle A., Raynaud F., Jackman A.L. (1996). Immunoreactive dUMP and TTP pools as an index of thymidylate synthase inhibition; effect of tomudex (ZD1694) and a nonpolyglutamated quinazoline antifolate (CB30900) in L1210 mouse leukaemia cells. Biochem. Pharmacol..

[58] Webley S.D., Hardcastle A., Ladner R.D., Jackman A.L., Aherne G.W. (2000). Deoxyuridine triphosphatase (dUTPase) expression and sensitivity to the thymidylate synthase (TS) inhibitor ZD9331. Br. J. Cancer.

[59] Costi M.P., Tondi D., Rinaldi M., Barlocco D., Pecorari P., Soragni F., Venturelli A., Stroud R.M. (2002). Structure-based studies on species-specific inhibition of thymidylate synthase. Biochim. Biophys. Acta.

[60] Forsthoefel A.M., Peña M.M., Xing Y.Y., Rafique Z., Berger F.G. (2004). Structural determinants for the intracellular degradation of human thymidylate synthase. Biochemistry.

[61] Peña M.M., Xing Y.Y., Koli S., Berger F.G. (2006). Role of N-terminal residues in the ubiquitin-independent degradation of human thymidylate synthase. Biochem. J..

[62] Forsthoefel A.M., Peña M.M., Xing Y.Y., Rafique Z., Berger F.G. (2004). Structural determinants for the intracellular degradation of human thymidylate synthase. Biochemistry.

[63] Peña M.M., Xing Y.Y., Koli S., Berger F.G. (2006). Role of N-terminal residues in the ubiquitin-independent degradation of human thymidylate synthase. Biochem. J..

[64] Houghton J.A., Houghton P.J. (1983). Elucidation of pathways of 5-fluorouracil metabolism in xenografts of human colorectal adenocarcinoma. Eur. J. Cancer Clin. Oncol..

[65] Peters G.J., Backus H.H., Freemantle S., van Triest B., Codacci-Pisanelli G., van der Wilt C.L., Smid K., Lunec J., Calvert A.H., Marsh S., McLeod H.L., Bloemena E., Meijer S., Jansen G., van Groeningen C.J., Pinedo H.M. (2002). Induction of thymidylate synthase as a 5-fluorouracil resistance mechanism. Biochim. Biophys. Acta.

[66] Priest D.G., Ledford B.E., Doig M.T. (1980). Increased thymidylate synthetase in 5-fluorodeoxyuridine resistant cultured hepatoma cells. Biochem. Pharmacol..

[67] Berger S.H., Barbour K.W., Berger F.G. (1988). A naturally occurring variation in thymidylate synthase structure is associated with a reduced response to 5-fluoro-2'-deoxyuridine in a human colon tumor cell line. Mol. Pharmacol..

[68] Jenh C.H., Geyer P.K., Baskin F., Johnson L.F. (1985). Thymidylate synthase gene amplification in fluorodeoxyuridine-resistant mouse cell lines. Mol. Pharmacol..

[69] Grem J.L., Fischer P.H. (1989). Enhancement of 5-fluorouracil's anticancer activity by dipyridamole. Pharmacol Ther..

[70] Chu E., Grem J.L., Johnston P.G., Allegra C.J. (1996). New concepts for the development and use of antifolates. Stem Cells.

[71] Wang W., McLeod H.L., Cassidy J., Collie-Duguid E.S. (2007). Mechanisms of acquired chemoresistance to 5-fluorouracil and tomudex: thymidylate synthase dependent and independent networks. Cancer Chemother. Pharmacol..

[72] Tajima A., Hess M.T., Cabrera B.L., Kolodner R.D., Carethers J.M. (2004). The mismatch repair complex hMutS alpha recognizes 5-fluorouracil-modified DNA: implications for chemosensitivity and resistance. Gastroenterology.

[73] Sasaki S., Watanabe T., Kobunai T., Konishi T., Nagase H., Sugimoto Y., Oka T., Nagawa H. (2006). hRFI overexpressed in HCT116 cells modulates Bcl-2 family proteins when treated with 5-fluorouracil. Oncol. Rep..

[74] Konishi T., Sasaki S., Watanabe T., Kitayama J., Nagawa H. (2006). Overexpression of hRFI inhibits 5-fluorouracil-induced apoptosis in colorectal cancer cells via activation of NF-kappaB and upregulation of BCL-2 and BCL-XL. Oncogene.

[75] Tseng Y.S., Tzeng C.C., Chiu A.W., Lin C.H., Won S.J., Wu I.C., Liu H.S. (2003). Exp. Cell Res..

[76] Brenes O., Arce F., Gätjens-Boniche O., Díaz C. (2007). Characterization of cell death events induced by anti-neoplastic drugs cisplatin, paclitaxel and 5-fluorouracil on human hepatoma cell lines: Possible mechanisms of cell resistance. Biomed. Pharmacother..

[77] Guo X., Goessl E., Jin G., Collie-Duguid E.S., Cassidy J., Wang W., O'Brien V. (2008). Cell cycle perturbation and acquired 5-fluorouracil chemoresistance. Anticancer Res..

[78] Lechner M., Lirk P., Rieder J. (2005). Inducible nitric oxide synthase (iNOS) in tumor biology: the two sides of the same coin. Semin. Cancer Bio..

[79] Romashkova J.A., Makarov S.S. (1999). NF-kappaB is a target of AKT in anti-apoptotic PDGF signalling. Nature.

[80] Islam S., Hassan F., Tumurkhuu G., Ito H., Koide N., Mori I., Yoshida T., Yokochi T. (2007). 5-Fluorouracil prevents lipopolysaccharide-induced nitric oxide production in RAW 264.7 macrophage cells by inhibiting Akt-dependent nuclear factor-kappaB activation. Cancer Chemother. Pharmacol..

[81] Shin Y.K., Yoo B.C., Chang H.J., Jeon E., Hong S.H., Jung M.S., Lim S.J., Park J.G. (2005). Down-regulation of Mitochondrial F1F0-ATP Synthase in Human Colon Cancer Cells with Induced 5-Fluorouracil Resistance. Cancer Res..

[82] Hwang I.T., Chung Y.M., Kim J.J., Chung J.S., Kim B.S., Kim H.J., Kim J.S., Yoo Y.D. (2007). Drug resistance to 5-FU linked to reactive oxygen species modulator. Biochem. Biophys. Res. Commun..

[83] Chu E., Zinn S., Boarman D., Allegra C.J. (1990). Interaction of γ interferon and 5-Fluorouracil in the H630 human colon carcinoma cell line. Cancer Res..

[84] Swain S.M., Boarman D., Allegra C.J. (1989). Fluorouracil and high-dose leucovorin in previously treated patients with metastatic breast cancer. J. Clin. Oncol..

[85] Ishii T., Marumo K. (2004). Biochemical modulation of 5-fluorouracil with interferonα/βandγon murine renal cell carcinoma. Int. J. Urol..

[86] Kang H.C., Kim I.J., Park H.W., Jang S.G., Ahn S.A., Yoon S.N., Chang H.J., Yoo B.C., Park J.G. (2007). Regulation of MDK expression in human cancer cells modulates sensitivities to various anticancer drugs: MDK overexpression confers to a multi-drug resistance. Cancer Lett..

[87] Yoo B.C., Jeon E., Hong S.H., Shin Y.K., Chang H.J., Park J.G. (2004). Metabotropic glutamate receptor 4-mediated 5-Fluorouracil resistance in a human colon cancer cell line. Clin. Cancer Res..

[88] Katakura K., Fujise H., Takeda K., Kaneko O., Torii M., Suzuki M., Chang K.P., Hashiguchi Y. (2004). Overexpression of LaMDR2, a novel multidrug resistance ATP-binding cassette transporter, causes 5-fluorouracil resistance in Leishmania amazonensis. FEBS Lett..

[89] Pratt S., Shepard R.L., Kandasamy R.A., Johnston P.A., Perry W., Dantzig A.H.  (2005). The multidrug resistance protein 5 (ABCC5) confers resistance to 5-fluorouracil and transports its monophosphorylated metabolites. Mol. Cancer Ther..

[90] Fanciullino R., Giacometti S., Mercier C., Aubert C., Blanquicett C., Piccerelle P., Ciccolini J. (2007). In vitro and in vivo reversal of resistance to 5-fluorouracil in colorectal cancer cells with a novel stealth double-liposomal formulation. Br. J. Cancer..

[91] Tsujie M., Nakamori S., Nakahira S., Takahashi Y., Hayashi N., Okami J., Nagano H., Dono K., Umeshita K., Sakon M., Monden M. (2007). Human equilibrative nucleoside transporter 1, as a predictor of 5-fluorouracil resistance in human pancreatic cancer. Anticancer Res..

[92] Park J.S., Young Yoon. S, Kim J.M., Yeom Y.I., Kim Y.S., Kim N.S. (2004). Identification of novel genes associated with the response to 5-FU treatment in gastric cancer cell lines using a cDNA microarray. Cancer Lett..

[93] Kim H.K., Choi I.J., Kim H.S., Kim J.H., Kim E., Park I.S., Chun J.H., Kim I.H., Kim I.J., Kang H.C., Park J.H., Bae J.M., Lee J.S., Park J.G. (2004). DNA microarray analysis of the correlation between gene expression patterns and acquired resistance to 5-FU/cisplatin in gastric cancer. Biochem Biophys Res. Commun..

[94] De Angelis P.M., Svendsrud D.H., Kravik K.L., Stokke T. (2006). Cellular response to 5-fluorouracil (5-FU) in 5-FU-resistant colon cancer cell lines during treatment and recovery. Mol. Cancer.

[95] Wang W., Cassidy J., O'Brien V., Ryan K.M., Collie-Duguid E. (2004). Mechanistic and predictive profiling of 5-Fluorouracil resistance in human cancer cells. Cancer Res..

[96] de Angelis P.M., Fjell B., Kravik K.L., Haug T., Tunheim S.H., Reichelt W., Beigi M., Clausen O.P., Galteland E., Stokke T. (2004). Molecular characterizations of derivatives of HCT116 colorectal cancer cells that are resistant to the chemotherapeutic agent 5-fluorouracil. Int. J. Oncol..

[97] Nordgard S.H., Alnaes G.I., Hihn B., Lingjaerde O.C., Liestøl K., Tsalenko A., Sørlie T., Lønning P.E., Børresen-Dale A.L., Kristensen V.N. (2008). Pathway based analysis of SNPs with relevance to 5-FU therapy: relation to intratumoral mRNA expression and survival. Int. J. Cancer..

[98] Ooyama A., Okayama Y., Takechi T., Sugimoto Y., Oka T., Fukushima M. (2007). Genome-wide screening of loci associated with drug resistance to 5-fluorouracil-based drugs. Cancer Sci..

[99] Schmidt W.M., Kalipciyan M., Dornstauder E., Rizovski B., Steger G.G, Sedivy R, Mueller M.W, Mader R.M. (2004). Dissecting progressive stages of 5-fluorouracil resistance in vitro using RNA expression profiling. Int. J. Cancer.

[100] Szöke D., Györffy A., Surowiak P., Tulassay Z., Dietel M., Györffy B. (2007). Identification of consensus genes and key regulatory elements in 5-fluorouracil resistance in gastric and colon cancer. Onkologie.

[101] Zhang F.M., Yao X.J., Tian X., Tu Y.Q. (2006). Synthesis and biological evaluation of new 4beta-5-Fu-substituted 4'-demethylepipodophyllotoxin derivatives. Molecules.

[102] Tian Z.Y., Du G.J., Xie S.Q., Zhao J., Gao W.Y., Wang C.J. (2007). Synthesis and bioevaluation of 5-fluorouracil derivatives. Molecules.

[103] Etienne M.C., Cheradame S., Fischel J.L., Formento P., Dassonville O., Renée N., Schneider M., Thyss A., Demard F., Milano G. (1995). Response to fluorouracil therapy in cancer patients: the role of tumoral dihydropyrimidine dehydrogenase activity. J. Clin. Oncol..

[104] Johnston P.G., Lenz H.J., Leichman C.G., Danenberg K.D., Allegra C.J., Danenberg P.V., Leichman L. (1995). Thymidylate synthase gene and protein expression correlate and are associated with response to 5-fluorouracil in human colorectal and gastric tumors. Cancer Res..

[105] Shirasaka T., Nakano K., Takechi T., Satake H., Uchida J., Fujioka A., Saito H., Okabe H., Oyama K., Takeda S., Unemi N., Fukushima M. (1996). Antitumor activity of 1 M tegafur-0.4 M 5-chloro-2,4-dihydroxypyridine-1 M potassium oxonate (S-1) against human colon carcinoma orthotopically implanted into nude rats. Cancer Res..

[106] Schöffski P. (2004). The modulated oral fluoropyrimidine prodrug S-1, and its use in gastrointestinal cancer and other solid tumors. Anticancer Drugs.

[107] Sakurai Y., Sakamoto K., Sugimoto Y., Yoshida I., Masui T., Tonomura S., Inaba K., Shoji M., Nakamura Y., Uyama I., Komori Y., Ochiai M., Matsuura S., Tanaka H., Oka T., Fukushima M. (2006). Blackwell Publishing Asia Orotate phosphoribosyltransferase levels measured by a newly established enzyme-linked immunosorbent assay in gastric carcinoma. Cancer Sci..

[108] Shi L.X., Ma R., Lu R., Xu Q., Zhu Z.F., Wang L., Zhou C.L., Li X.L., Zhang H.L., Yao Z. (2008). Reversal effect of tyroservatide (YSV) tripeptide on multi-drug resistance in resistant human hepatocellular carcinoma cell line BEL-7402/5-FU. Cancer Lett..

[109] Yu Z.W., Zhao P., Liu M., Dong X.S., Tao J., Yao X.Q., Yin X.H., Li Y., Fu S.B. (2004). Reversal of 5-flouroucial resistance by adenovirus-mediated transfer of wild-type p53 gene in multidrug-resistant human colon carcinoma LoVo/5-FU cells. World J. Gastroenterol..

[110] Zhu H., Guo W., Zhang L., Davis J.J., Teraishi F., Wu S., Cao X., Daniel J., Smythe W.R., Fang B. (2005). Bcl-XL small interfering RNA suppresses the proliferation of 5-fluorouracil-resistant human colon cancer cells. Mol. Cancer Ther..

[111] Zhu H., Zhang L., Huang X., Davis J.J., Jacob D.A., Teraishi F., Chiao P., Fang B. (2004). Overcoming acquired resistance to TRAIL by chemotherapeutic agents and calpain inhibitor I through distinct mechanisms. Mol. Ther..

[112] Mikano G., Etienne M.C., Pierrefite V., Barberi-Heyob M., Deporte-Fety R., Renée N. (1999). Dihydropyrimidine dehydrogenase deficiency and fluorouracil-related toxicity. Br. J. Cancer.

[113] Raida M., Schwabe W., Häusler P., Van Kuilenburg A.B., Van Gennip A.H., Behnke D., Höffken K. (2001). Prevalence of a common point mutation in the dihydropyrimidine dehydrogenase (DPD) gene within the 5’-splice donor site of intron 14 in patients with severe 5-fluorouracil (5-FU)-related toxicity compared with controls. Clin. Cancer Res..

